# Threatened Death during Major Anesthesia

**Published:** 1897-09

**Authors:** Robert H. M. Dawbarn

**Affiliations:** Professor of Operative Surgery and of Surgical Anatomy, New York Polyclinic


### THREATENED DEATH DURING MAJOR ANESTHESIA.*

By ROBERT H. M. DAWBARN, M.D.,

Professor of Operative Surgery and of Surgical Anatomy, New York Polyclinic.

There are two surgical emergencies so appalling in their character, and so quickly fatal if not promptly met, that they may be said to transcend in importance all others. These are, anesthesia-narcosis threatening death, and unexpected hemorrhage upon the operating-table. In each of these the safety of the patient demands that the surgeon shall have his knowledge at his finger-tips, and be ready instantly and almost automatically to do the right thing.

*Continued from August number. Dr. Dawbarn wished to insert the following notes in his article in our August issue; they arrived too late, however, for this purpose:

[Prof. R. Ogden Doremus informs the writer that many years ago in a lecture at Steinway Hall, N. Y., for the benefit of the widow of Dr. Horace Wells, Dr. J. Marion Sims stated that he (Sims) had performed an operation an hour and twenty minutes long under laughing-gas anesthesia.

It would seem an obvious advantage and great convenience if laughing-gas mingled with 10 per cent. of oxygen could be liquefied together and supplied from the same canister to the patient. This is not feasible, however. Oxygen requires 300 atmospheres (more than two tons to the square inch) plus the most intense cold to make it liquid, and would hardly be safe in a portable canister. Laughing-gas needs but 40 atmospheres for liquefaction, as stated heretofore.—R. H. M. D.]

The first of these is the topic under discussion in this paper; and no one will demur to the statement that it is the duty of every doctor to memorize the measures to meet this indication until they are as familiar to him—as much a part of himself—as is his alphabet. For no time will be left to him for debate when confronted by the actual fact. Every moment is then golden, and indecision equally with unwise choice may cost a life.

*Prevention.*—Prevention is half the battle. If the suggestions under this heading are heeded, few indeed will be the actual cases encountered.

The writer makes it an invariable rule, and has done so for many years, to precede every exhibition of either chloroform or ether by a hypodermic injection of morphine sulphate guarded by atropine sulphate; in the adult from grain 1-4 to grain 1-3 of the former, and from grain 1-150 to grain 1-120 of the latter; allowing, say, a quarter of an hour for it to take effect. Also as a rule, an ounce or more of whisky or brandy is given at the same time.

There are several reasons for this course, but one alone should suffice; namely, that all of these drugs are analgesics, and that consequently much less of the anesthetic need be inhaled, and the danger of death is just to that extent lessened.

Indeed, if any reason exists why neither ether nor chloroform nor laughing-gas should be taken, a fairly comfortable operation is possible by free use of these means alone, as all surgeons know.

Again; nearly 50 per cent. of all deaths from chloroform have occurred before complete anesthesia was accomplished; commonly within the first minute or two of its exhibition, and when only a very little had as yet been used.* Had the stage of excitement and of fright been prevented by a little morphine, with its calmative and steadying influence on the reflexes, this mortality would probably have been avoided.

It will be noted that all three of the aids mentioned (morphine, atropine, alcohol) are heart-tonics when used in moderation, and tend also to diminish the likelihood of operative shock; and further, atropine is one of our best vasomotor stimulants.

*Wood's Ref. Handbook, *Anesthetics*, Vol. 1, p. 100.

(For all the reasons mentioned in the last sentence the writer approves of the use of strychnine sulphate, in small doses, for a few days prior to any severe operation. Perhaps, when shock is actually present, it is of more value than any other one drug; and if this be true, its use in *prevention* of shock would seem sensible.)

Dr. H. C. Wood has demonstrated the value of strychnine when used by needle in treating anesthesia-narcosis; and here again its use in *prevention* seems occasionally indicated.

Although we approve of the use of alcohol *prior* to anesthesia, as just stated, because then less of the anesthesia will be needed, a clear distinction must here be drawn between this fact and its exhibition as a remedy where death is actually threatening from an excess of ether or chloroform. This distinction must not be forgotten.

The writer has witnessed again and again instances of the accident under discussion, in which the operator and his assistants endeavored by numerous hypodermic injections of whisky or brandy —narcotics—to revive a patient suffering from an excess of another narcotic. Dr. H. C. Wood has used the strong language to characterize this procedure, that "it is little better than murder!" To be sure, alcohol is a heart-tonic, but we have other equally swift heart-tonics which do *not* tend to deepen the deadly narcosis.

Rather recently the cocainization of the nares and larynx by spray, just prior to anesthesia, has been considerably used. It certainly renders the inhalation less unpleasant, and in the case of chloroform, may quite conceivably prevent danger. We know that very early in a chloroform-anesthesia a certain danger sometimes exists that this irritant drug may cause a reflex action whereby the pneumogastric nerve stops the heart—and it may not wake up again.*

Quite a little work has lately been done by various surgeons regarding anesthesia by ether mixed with oxygen. It would seem that the idea may be of value in helping to avoid narcosis in excess. It has not, however, come into general use. It is a revival, in principle, at least, of the "ozonic ether" of Dr. Richardson, who

*The suggestions in the opening paragraphs under *prevention* form an effective safeguard against this danger.

advised this for anesthesia before the Brit. Med. Association in 1865. He produced this compound by agitating a 30-volume solution of peroxide of hydrogen with anhydrous ether, and adding 5 per cent. of alcohol.

Under the heading of prevention we must remember not to begin cutting until the patient is "under to the surgical degree." Buxton, Reeve and other writers upon anesthetics have pointed out the surprisingly large proportion of cases in which incomplete anesthesia, by lowering vital functions, has permitted fatal shock to result from trivial though painful operations, such as strabismus, tooth-pulling, circumcision, etc. Indeed, it is estimated that 40 per cent. of chloroform deaths have been in cases of such a trifling nature, and there seems no other adequate explanation of so large a proportion in comparison with deaths during major operations.

Again, in reference to chloroform, it is probably safer to tip the table so as to have the head a trifle lower than the feet; this as a measure of precaution, and for reasons to be more fully discussed later, under the heading of *treatment*. But the patient may be permitted to choose whether to lie upon the back or the side during the inhalation.

Many are the recorded cases of death from chloroform exhibited in the sitting or the half-reclining postures; which invite anemia of the vital centers. Dr. H. A. Hare has recently pointed out the fact that chloroform kills really by vasomotor paralysis, whereby "the man is suddenly bled into his own veins and capillaries, as effectively as if into a bowl"; and instant anemia of brain and heart results.

It is the part of wisdom to insist upon twenty-four hours, at least, in hospital (preferably) and in bed, before anesthesia. Bodily rest and time in which the mind may grow accustomed to the new environment are of obvious value.

A rule, which should be inflexible, requires the cautious surgeon to make a physical examination of his patient's various organs shortly before operating. Notably, the kidneys must not be neglected. Presence of albumin should interdict the use of ether. It is believed that those with impaired renal function are more apt than others to succumb to the narcosis. Certain surgeons, and especially Dr. R. F. Weir, consider chloroform at least equally an irritant to the kidneys. Here we surely have an indication for the employment of nitrous oxide anesthesia.

If any faintness be noted due to the preparatory fasting which we must enforce, nothing but benefit can result from a nutrient enema an hour or so before the operation. This may also contain whisky or brandy if desired, and is a good way in which to exhibit the alcohol which we have advised for routine use in preparation for anesthesia.

Perhaps it is a waste of words to mention that the bowels and bladder must be recently evacuated, and that it is a duty of the anesthetist to see that corsets and all tight clothing are removed, also artificial teeth. Let the inhalation be most gradually commenced; it is always a mistake to force a sudden unconsciousness. With a nervous patient grant permission, if desired, to hold the inhaler himself for a time; and meanwhile a few cheery words will often allay fright, which is dangerous.

The beginner should be taught that the palpebral reflex is practically as good a guide as the conjunctival. There is no justification for the subsequent sore eyes due to frequent rubbing of the cornea or conjunctiva often to be noted.

The writer considers stertorous respiration (which is due to paralysis of the *velum pendulum palati*, from deep anesthesia) a sufficient indication, as a rule though not invariably, for permitting inhalation of a larger proportion of air. To keep the patient snoring heavily through a long operation is to subject him to a nearer approach to a lethal dose than the writer deems wise.

The color of the blood is to be noted by the surgeon, and if it seems a shade too dark the anesthetist should be warned. The pupils too are a fair though not quite reliable guide. Should they suddenly dilate it means instant peril.

In using a tampon-canula after tracheotomy, for exhibition of the anesthetic, let it be remembered that here especial care is needed, as it is unpleasantly easy to exclude all oxygen, and to have an asphyxiated patient in consequence.

Perhaps a word may not be amiss regarding anesthesia by rectum. This is practicable by ether-vapor, the bottle being placed in hot water and the vapor conveyed by a rubber tube *upwards* into the rectum. *Upwards*, to avoid possibility of pouring fluid ether into the bowel. The advantage is, avoidance of the excitement, coughing and semi-strangling sometimes observed while beginning the ordinary method; also it is of use where actual cautery about the mouth or nose is contemplated. Disadvantages are occasional causation of a severe proctitis; and also in event of excessive narcosis, the patient, although the supply be stopped, must absorb the large amount of ether-vapor filling his rectum and colon before he can begin to revive.

When a child is to be anesthetized, chloroform being the intended means, it is an excellent plan to avoid all excitement and fright by giving it during sleep. With a moderate amount of skill this is not a difficult feat, the child gently sliding from the natural into the artificial slumber. With adult patients, however, while possible of accomplishment in occasional instances, this is not generally practicable.

And finally, under *prevention* still, we should allude to a plan, little advocated in print, but which has only to be witnessed once in order to commend itself. Namely, as a measure of precaution (in a patient of feeble vitality about to be subjected to operation), *maintaining a reserve-guard of pure blood*, by accumulating it in one or more of the extremities; to be released, in event of need, during narcosis.

For other purposes this temporary sequestration of blood has been employed by a number of physicians; but Dr. David Webster was first to suggest it for *this* indication, in 1887, in a letter to the *N. Y. Med. Journal;* and shortly thereafter the writer was the first actually so to use it.

The technique is simple. The thigh, for example, is corded at the groin; at first slightly, for a few minutes, permitting it to swell visibly, from accumulated blood, and then tightly enough to obstruct all flow in either direction. This is done with a rubber bandage. The limb is now kept wrapped in a blanket with warm bottles or bricks about it. In case of dangerous narcosis, release the band, elevate the limb, and you pour great quantities of pure blood into the body surcharged with ether or chloroform. The good results are striking and supervene promptly.

It is obvious that in an operation which will probably occupy much time this plan might not be wise; but for half an hour or less the blood could be so retained without danger to the limb.*

Last, under the heading of prevention, but not least, we should mention the advisability of placing on guard at the head of the operating-table only an experienced man. In almost every hospital this work is relegated to the junior assistant—in the opinion of the writer, a grave mistake. If that junior has had instead some months during which opportunity is given him to observe the proper methods and learn to avoid the perils of anesthesia, then as senior assistant he becomes a comparatively safe man to trust.

*Treatment.*—Dr. H. C. Wood, in his study of this subject for the Berlin Congress, teaches that the treatment should be practically identical whether for ether or for chloroform-narcosis; and this is the doctrine we will follow.

Under this heading, in order to be systematic and orderly, the better to aid memorizing, let us discuss:

1. Respiratory trouble. (*a*) Obstruction to the entrance of air. (*b*) Failure of the respiratory center.

2. Heart trouble.

Of course, too, we may have to treat both 1 and 2 at the same time.

### 1. RESPIRATORY TROUBLE.

Under (*a*) of the first head we have to deal with dropping of the tongue back upon the larynx, causing asphyxia; and also with obstruction from thick, ropy mucus, or blood, or pus, or vomited food inhaled.

Trouble with the tongue is best met by Benjamin Howard's method of raising the epiglottis. This can rarely be done effect-

*For fuller details, and a study of the other purposes for which, prior to an operation, this plan may be employed, the reader is referred to the writer's article upon Anesthesia Narcosis, in Vol. IX. of Wood's Reference Handbook, and in which is quoted in full an essay by Dr. Charles McBurney upon this point.

tively by use of tongue-forceps (though these may of course be tried), and if such traction fail to relieve the dyspnea, the Howard position should be adopted, *by over-extending the head.* Turn it as sharply backward as possible, at the same time *closing* his mouth. You have now an almost straight passage from the nostrils to the larynx; and the tense geniohyoid muscles, and in turn the hyo-epiglottic ligaments, force the epiglottis to stand upright and to ward off the tongue from falling back upon the laryngeal entrance.

Instead of this plan, and somewhat less effectual, is the method of forcing the lower jaw forward by the thumbs of the anesthetizer placed behind the rima. Thereby again the above-named muscles and ligaments are made somewhat tense, also the genio-hyo-glossi muscles, and in turn the glossi-epiglottic attachments; and the epiglottis is thus lifted.

Should our difficulty be with foreign matter in the pharynx and mouth, here we use sponges on long holders. Often the gag meanwhile is necessary. Vomiting implies insufficient anesthesia, and a tendency to vomit should be met promptly and prevented by pushing the anesthetic until this reflex is subdued.

One need hardly remark that the operator imperils his patient's life who does not insist upon five or six hours' prior abstinence from food. But occasionally a patient disobeys the injunction and does not confess. Then too, we occasionally, if rarely, meet a case where from previous muscular atony of the stomach, or from obstruction at the pylorus, or from nervous gastric inactivity due to fear of the coming event, food eaten even twelve and twenty-four hours earlier is vomited during the operation.

Should a portion of this be inhaled, it will probably catch in the rima glottidis. In that case, inverting and shaking the patient must promptly be tried; and if imminent suffocation compels, the operation of laryngotomy must be done, which takes less time than the reading of this sentence. It is a single, transverse thrust through skin and crico-thyroid membrane, entering the air-space well below the chink of the glottis.

Under (*b*) we may try:

1. Laborde's method of rhythmic traction upon the tongue, quite vigorously, and about as frequently as the normal respiratory act.

2. Dr. W. Gill Wylie's plan, which is to seize a handful of the belly-wall, about the region of the navel, and to lift it sharply forward, away from the spinal column. At once the patient gasps from reflex stimulation. This is repeated again and again until normal respiration is established.

3. Stretching the sphincter ani vigorously with the thumbs.

4. Ice slipped into the rectum, or placed against the nape of the neck.

5. Slapping the chest and abdomen with towels wet in ice-water.

If the patient be "under to the surgical degree" it is plain that such methods as the foregoing, dependent as they are for success upon reflex stimulation, cannot be really effective until a portion of the anesthetic is eliminated from the blood and the reflexes return. Hence the use of:

6. Various methods of artificial respiration, such as Sylvester's, Hall's, Schultze's (with little children), and several more. Those just named are too well-known to need description. We may say a word, however, as to the technique of certain others. Kœnig's plan is intermittent pressure with both hands about the chest, one of the thumbs being placed in a space *over the heart* (differing simply in this latter respect from von Nussbaum's method) between the apex-beat and the sternum. And Maas's modification of Kœnig's, is one whereby the pressure aforesaid is made very rapidly —one hundred and twenty times a minute—instead of at the ordinary respiratory rate of speed.

All of these methods would seem to act mainly by frequent expulsion of narcotized air, and admission of fresh; though Kœnig and Maas claim for the thumb in one of the precordial spaces a mechanical stimulant or irritant effect upon the heart, as well.

7. Insertion of a soft catheter down the larynx and trachea, and use of the operator's lungs to replace narcotized air by fresh.

8. The bellows-method: A special and ingenious mechanism for removing exhausted or poisoned air, and replacing it at each stroke by an equal bulk of pure atmospheric air.

9. Inhalation of oxygen, while efforts at respiration are being artificially maintained. Every well-equipped operating-room should be supplied with a canister of oxygen, against such an emergency.

10. *Drugs.* Of these, atropine and strychnine, both by needle, are our chief reliance. Both have the value, beside being active respiratory stimulants, of being also vasomotor tonics.

(*To be concluded.*)

